# Prevalence and predictors of hypertension in Namibia: A national-level cross-sectional study

**DOI:** 10.1371/journal.pone.0204344

**Published:** 2018-09-20

**Authors:** Leslie S. Craig, Anastasia J. Gage, Albertina M. Thomas

**Affiliations:** 1 Department of Global Health Management and Policy, Tulane University School of Public Health and Tropical Medicine, New Orleans, Louisiana, United States of America; 2 Department of Global Community Health and Behavioral Sciences, Tulane University School of Public Health and Tropical Medicine, New Orleans, Louisiana, United States of America; 3 Ministry of Health and Social Services, Windhoek, Namibia; University of L’Aquila, ITALY

## Abstract

**Background:**

Hypertension has been identified as the single greatest contributor to the global burden of disease and mortality, with estimates suggesting that the highest levels of blood pressure have shifted from high-income countries to low-income countries in sub-Saharan Africa. While evidence suggests a remarkably high prevalence of hypertension among urban residents in Namibia, national estimates to inform on the country-level burden are lacking. This study estimates the prevalence and predictors of hypertension among Namibian adults.

**Methods:**

The analysis is based on 1,795 women and 1,273 men aged 35–64 years from the nationally-representative 2013 Namibia Demographic and Health Survey. Odds radios and 95% confidence intervals were estimated using logistic regression.

**Results:**

The age-standardized prevalence of hypertension was 46.0% (men vs. women: 46.1% vs. 46.0%). Mean systolic and diastolic blood pressures for the total population were 128.8 mmHg (95% CI 127.8–129.7) and 83.1 mmHg (95% CI 82.5–83.7), respectively. Mean systolic blood pressure was significantly lower among women (men vs. women: 130.9 mmHg vs. 127.4 mmHg; *p*<0.001). There were no statistically significant differences in mean diastolic blood pressure between men and women. Older age, urban residence, and being either overweight or obese were positively associated with the odds of hypertension (p<0.01). For women, the odds of hypertension were also significantly increased for those who were diabetic (i.e. had a fasting blood glucose level greater than 7.0 mmol/L) and reduced for those with higher levels of education.

**Conclusion:**

The prevalence of hypertension among Namibian adults is high and associated with metabolic and socio-demographic factors. Future research examining disease comorbidity and behavioral risk factors could better inform on the disease burden and help target resources to optimize prevention and control.

## Introduction

Raised blood pressure, or hypertension, has been identified as the greatest single contributor to the global burden of disease and mortality, with an effect that is largely mediated through coronary heart disease and stroke, and noted to be responsible for 9.4 million deaths each year [1–4]. Previously considered a disease of low prevalence in the developing world, evidence suggests that hypertension is of greater economic burden to developing countries than developed ones [2,4]. A pooled analysis of worldwide trends in blood pressure from 1975 to 2015 shows that the highest levels of blood pressure worldwide have shifted from high-income countries to low-income countries in South Asia and sub-Saharan Africa (SSA) [3]. This changing circumstance is of great concern in SSA, given the dual burden of communicable and non-communicable diseases (NCDs) with which many resource-constrained countries in the region must now contend [2,4–7]. The situation is aggravated by the increased risk of adverse outcomes, primarily due to lower levels of hypertension awareness and control, a younger age of onset of the disease, and a potentially more aggressive disease course observed in persons of black African ancestry [1,2,8–11].

Although evidence of an escalating pattern of hypertension in SSA has been demonstrated in the burgeoning amount of research on hypertension in this region [8–12], experts believe the real burden is still far from known, due to limited, heterogenous or contradictory data, frequent use of non-national samples, and inclusion of only some countries and some regions [3,8,12,13]. For example, while the World Health Organization (WHO) reports the estimated prevalence of hypertension in the African Region in 2008 to be the highest globally at 46% [14], estimates from other studies are relatively lower [4,12,13]. Several variations have also been reported regarding sex differences in hypertension prevalence. For instance, a 2007 systematic review of 25 studies across 10 countries in SSA identified minimal differences in hypertension by sex, concluding that urban residence and advancing age were the most significant determinants of higher prevalence [8]. In comparison, a 2011 review using data from 15 studies across 11 countries in SSA, cited higher prevalence of hypertension in men than in women, noting that the sex differences tended to be more pronounced in urban than in rural areas, though not statistically significant [12]. Data from 2017 pooled analyses, on the other hand, suggest that both mean systolic and diastolic blood pressures have increased among men and women in SSA, and that a gender differential exists in the burden of disease, with a female excess in age-standardized mean systolic blood pressure (SBP) and hypertension prevalence [3].

Accordingly, currently available literature consistently underlines the need for more nationally representative surveys to better elucidate the country-level burden of hypertension and the problem it presents for the SSA region [3,4,8–10,12]. This need for national-level data on hypertension burden appears particularly relevant given reports that the prevalence of hypertension in some SSA countries is among the world’s highest [8,11,13]. Indeed, a 2012 cross-sectional study of hypertension across four rural and urban communities in SSA reported age-standardized prevalence estimates of 19.3% (95%CI:17.3–21.3), 21.4% (19.8–23.0), 23.7% (21.3–26.2) and 38.0% (35.9–40.1) in rural Nigeria, rural Kenya, urban Tanzania and urban Namibia, respectively [15], supporting theories of the magnitude of the hypertension burden in SSA and providing shocking evidence of a remarkably high burden among urban Namibian residents (crude: 32.0%; age-standardized: 38.0%) similar to that of non-Hispanic black adults in the USA (38.6%) [16,17]. Despite evidence of high disease prevalence in this urban population, however, no studies have yet addressed the national burden of hypertension in Namibia.

Namibia is a middle-income country in SSA with a population just over 2 million [18,19]. The population is described as young, with 57.8% under 25 years of age and only 3.5% aged 65 years or older [20]. One of the least densely populated countries in the world, Namibia is divided into 14 administrative regions and, despite rapid urbanization, remains largely rural [18,19]. While infectious diseases remain prevalent in the population, WHO estimates suggest that NCDs are of significant concern to national morbidity and mortality [21]. As a major modifiable risk factor for NCDs, knowledge of the prevalence of hypertension, combined with increased awareness, treatment, and control of the condition, present a powerful means of predicting risk, preventing disease, and avoiding death [4]. Nationally representative data from the 2013 Namibia Demographic and Health Survey (NDHS) are now available and include physical and biochemical measurements of common NCD biomarkers (e.g. blood pressure, fasting blood glucose, body mass index (BMI)) among a subsample of women and men, 35–64 years of age. This study aims to estimate the prevalence of hypertension among Namibian adults 35–64 years of age, and the associations with select socio-demographic, metabolic and behavioral factors, in order to provide baseline data on the hypertension burden in country and inform the targeting of interventions to improve overall management and prevention of the disease.

## Methods

This study employed secondary analysis of data from the 2013 NDHS, the fourth comprehensive, national-level population and health survey conducted in Namibia as part of the global Demographic and Health Surveys (DHS) program [18]. The 2013 NDHS used a two-stage stratified cluster design and was the first national survey in Namibia to include biomarker measurements of blood pressure and fasting blood glucose [18]. Via the Household Questionnaire, self-reported health status and biomarker data were collected among eligible men and women 35–64 years of age, in half of the surveyed households.

To assess blood pressure, three measurements were taken using the Life Source UA-767 Plus digital device with automatic upper-arm inflation and automatic pressure release, ensuring a minimum five-minute resting interval between readings. The first measurement was typically discarded and the average of the last two measurements used as the blood pressure level of the participant [18]. However, if consent was only given for two readings, the second reading was taken as the average while, if there was only one blood pressure reading, it was recorded as the average [18]. For the fasting blood sugar test, a blood sample was obtained via finger prick, following an overnight fast, and tested using the HemoCue Glucose 201 RT system (HemoCue Ab, Angelholm Sweden) to determine blood glucose levels [18]. In addition to these biomarker assessments, self-reported data were collected using survey language from the WHO “STEPwise approach to the surveillance of non-communicable diseases” (STEPS) instrument and the World Health Survey 2003 questionnaire [18,22]. Specifically, participants were asked whether they had ever been told by a doctor or other health worker that they have high blood pressure (or hypertension), or high blood sugar (or diabetes), and those who responded affirmatively were then asked whether they had been prescribed any medication to control the disease. Further details of the survey design, sampling procedures and data collection methods are provided in the country report [18].

Analyses in this paper were restricted to participants with non-missing information on key variables of interest (i.e. sex, age, education level, ethnicity, place of residence, employment status, wealth quintile, smoking, BMI, fasting blood sugar levels, and systolic and diastolic blood pressure measurements). Participants were additionally excluded if they were missing self-reports of hypertension or diabetes diagnosis. Of the 4,747 surveyed respondents (2,163 men and 2,584 women) aged 35–64 years, 47 pregnant women (1.0%) were excluded since hypertension in pregnancy has a different etiology [23]. Of the remaining 4,700 participants, 1,113 (23.7%) were missing blood pressure measurements. These blood pressure measurements were missing due to refusals (17.4%), the respondent being absent from the home (30.1%), technical problems (0.2%), other problems (1.5%) and non-response (50.8%). Coverage rates for blood pressure measurement among men and women were 71.2% and 80.7%, respectively. Among the 3,587 remaining participants (1,539 men and 2,048 women) with valid blood pressure measurements, 519 (14.5%) were missing information on one or more socio-demographic and/or risk factor variables. Respondents who were excluded from the analysis due to missing data tended to be younger (*p*<0.001), male (*p*<0.001), currently working (*p*<0.001), in the richer or richest quintiles (*p*<0.001), have lower levels of education(*p*<0.001), and live in urban regions (*p*<0.001). These differentials must be borne in mind in interpretation of findings. The final analytic sample of 3,068 included 1,795 eligible women and 1,273 eligible men, 35–64 years of age.

### Ethics

Ethical approval of the survey instruments and procedures was granted by the Ministry of Health and Social Services (MoHSS) Biomedical Research Committee, the Institutional Review Board of ICF International and the U.S. Centers for Disease Control and Prevention. Written consent was obtained from all participants prior to the interview and data were collected confidentially.

### Statistical analysis

#### Variables

The primary outcome in this study was hypertension prevalence, which was defined, according to WHO criteria as SBP of 140 mmHg or greater, diastolic blood pressure (DBP) of 90 mmHg or greater, and/or currently taking antihypertensive medications [14]. Pre-hypertension was defined as an SBP of 120–139 mmHg and/or a DBP of 80 to 89 mmHg among persons not on treatment for hypertension. Individuals with a normal blood pressure reading who self-reported a previous diagnosis of high blood pressure by a doctor or other health worker but were not on treatment were not considered hypertensive.

Additional analyses also explored variation in secondary outcomes of hypertension awareness, treatment and control by socio-demographic characteristics. Awareness of hypertension was defined according to self-report of previous diagnosis of high blood pressure (hypertension) by a doctor or other health worker, among participants identified as having hypertension. Treatment of hypertension was defined as self-reported use of prescribed medication to control blood pressure among those who self-reported to have hypertension. Control of hypertension was defined as pharmacologic treatment of hypertension associated with an average SBP <140 mm Hg and an average DBP <90 mm Hg among those who self-reported to have hypertension.

Independent variables were coded categorically and included the sex of the respondent (male or female), age-group (35–39, 40–44, 45–49, 50–54, 55–59 or 60–64 years), highest level of education attended (no education or preschool only, primary schooling, or secondary school or higher), place of residence (urban or rural), and quintiles of wealth. The NDHS 2013 did not explicitly allow for self-report of ethnicity so language of the respondent was used as a proxy (Oshiwambo, Damara/Nama, Afrikaans, Herero, or other). Occupational status was defined as not currently working or currently working, with the latter category further classified using the International Standard Classification of Occupations (ISCO-08). Major ISCO-08 groups were collapsed to create three categories: managerial/professional/technical/clerical, sales/services/industry, and other. The other category included those in elementary occupations, armed forces occupations and those who indicated that they were currently working but did not specify their employment type. Current smoking status was classified into three categories (does not currently smoke, smokes cigarettes, or smokes pipes, cigars, etc.). BMI was classified based on WHO categories of underweight (less than 18.5 kg/m^2^), normal weight (18.5–24.9 kg/m^2^), overweight (25.0–29.9 kg/m^2^) and obese (≥30 kg/m^2^) [14]. Diabetes was defined as having a fasting plasma glucose value ≥ 7.0 mmol/L (126 mg/dl) or being on medication for raised blood glucose [14].

#### Data analysis

Descriptive statistics and frequency distributions were used to describe participant characteristics. Prevalence data were weighted to account for the sampling design and non-response. Age-standardized prevalence estimates of hypertension were also calculated using the WHO world standard population [24] by taking weighted means of age-sex-specific estimates, with use of age weights from the standard population. In separate analyses by sex, odds radios (ORs) and 95% confidence intervals (CIs) for predictors of hypertension were estimated using logistic regression analyses. In accordance with recommended research practice, however, regressions were unweighted. All data were analyzed using Stata version 12 (StataCorp., College Station, TX, USA) and statistical significance accepted when *p*<0.05.

## Results

### Sample characteristics

Subject characteristics by sex are summarized in Table 1. The mean age of participants was 46.8 years. Compared to men, fewer women in the sample lived in urban areas (50.0% vs. 44.3%; *p*<0.01), were currently working (61.7% vs. 37.2%; *p*<0.001), lived in households in the middle to highest quintiles of wealth (65.9% vs. 59.5%; *p*<0.001) or smoked (26.7% vs. 10.3%; *p*<0.001). More women were, however, overweight (24.2% vs. 18.0%; *p*<0.001) and obese (24.6% vs. 10.1%; *p*<0.001).

**Table 1:** Characteristics of respondents aged 35–64 years, by sex (Namibia DHS 2013).

	Men (n = 1,273)	Women (n = 1,795)	Total (N = 3,068)	*p*-value
**Mean age (95% CI), years**	46.5 (46.0–47.1)	46.9 (46.5–47.4)	46.8 (46.4–47.2)	0.230
**Age-group, years**				
35–39	318 (25.7%)	422 (24.3%)	740 (24.8%)	0.116
40–44	285 (22.0%)	358 (20.2%)	643 (20.9%)	
45–49	229 (18.1%)	326 (17.4%)	555 (17.6%)	
50–54	174 (13.4%)	316 (18.0%)	490 (16.1%)	
55–59	137 (10.7%)	195 (10.1%)	332 (10.3%)	
60–64	130 (10.2%)	178 (10.1%)	308 (10.2%)	
**Education**				**0.011**
No education/preschool only	231 (16.8%)	241 (12.9%)	472 (14.4%)	
Primary	402 (31.7%)	638 (35.7%)	1,040 (34.1%)	
Secondary or higher	640 (51.5%)	916 (51.5%)	1,556 (51.5%)	
**Ethnicity**				**<0.001**
Oshiwambo	463 (45.8%)	758 (53.6%)	1,221 (50.5%)	
Damara/Nama	230 (12.9%)	295 (11.1%)	525 (11.8%)	
Afrikaans	201 (12.0%)	239 (9.4%)	440 (10.4%)	
Herero	148 (9.9%)	179 (7.8%)	327 (8.6%)	
Other	231 (19.4%)	324 (18.2%)	555 (18.7%)	
**Residence**				**0.003**
Rural	659 (50.0%)	981 (55.7%)	1640 (53.5%)	
Urban	614 (50.0%)	814 (44.3%)	1428 (46.5%)	
**Occupational status**				**<0.001**
Not currently working	469 (38.3%)	1,137 (62.8%)	1,606 (53.1%)	
Managerial/professional/ technical/clerical	131 (11.1%)	215 (13.0%)	346 (12.3%)	
Sales/services/industry	362 (29.5%)	121 (7.2%)	483 (16.0%)	
Other	311 (21.2%)	322 (16.9%)	633 (18.6%)	
**Wealth quintile**				**0.013**
Lowest	196 (17.5%)	332 (20.6%)	528 (19.4%)	
Second	222 (16.7%)	343 (19.8%)	565 (18.6%)	
Middle	261 (20.5%)	340 (18.8%)	601 (19.5%)	
Fourth	309 (23.5%)	411 (21.2%)	720 (22.1%)	
Highest	285 (21.9%)	369 (19.6%)	654 (20.5%)	
**Current smoking status**				**<0.001**
Does not currently smoke	882 (73.3%)	1,564 (89.7%)	2,446 (83.2%)	
Smokes cigarettes	309 (20.8%)	131 (4.9%)	440 (11.2%)	
Smokes pipes, cigars, etc.	82 (5.9%)	100 (5.4%)	182 (5.6%)	
**BMI status, kg/m**^**2**^				**<0.001**
Underweight	186 (15.7%)	156 (8.8%)	342 (11.5%)	
Normal weight range	723 (56.2%)	699 (42.5%)	1,422 (47.9%)	
Overweight	228 (18.0%)	447 (24.2%)	675 (21.7%)	
Obese	136 (10.1%)	493 (24.6%)	629 (18.8%)	
**Diabetes**	89 (6.6%)	120 (5.8%)	209 (6.2%)	0.466
**Mean SBP (95% CI), mmHg**	130.9 (129.3–132.4)	127.4 (126.2–128.6)	128.8 (127.8–129.7)	**<0.001**
**Mean DBP (95% CI), mmHg**	82.7 (81.7–83.6)	83.4 (82.6–84.2)	83.1 (82.5–83.7)	0.197
**Blood pressure status**				
Normal blood pressure	332 (26.6%)	465 (27.2%)	797 (27.0%)	0.838
Pre-hypertension	385 (29.1%)	517 (27.9%)	902 (28.4%)	
Hypertension	556 (44.3%)	813 (44.9%)	1,369 (44.6%)	

Note: Values are Unweighted N (Weighted %) or mean (95% CI)

### Prevalence of hypertension

The prevalence of hypertension was similar among women and men (crude: 44.6%; men: 44.3%; women: 44.9%). Mean SBP and mean DBP for the total population were 128.8 mmHg and 83.1 mmHG, respectively. Mean SBP was 3.5 mmHG lower for women than men (*p*<0.001). There were no significant differences in mean DBP between men and women (82.7 mmHG vs. 83.4 mmHg). Notably, while just over a quarter of the population was normotensive (27.0%), nearly 30% (men: 29.1%; women: 27.9%) were classified as pre-hypertensive.

**Fig 1:**
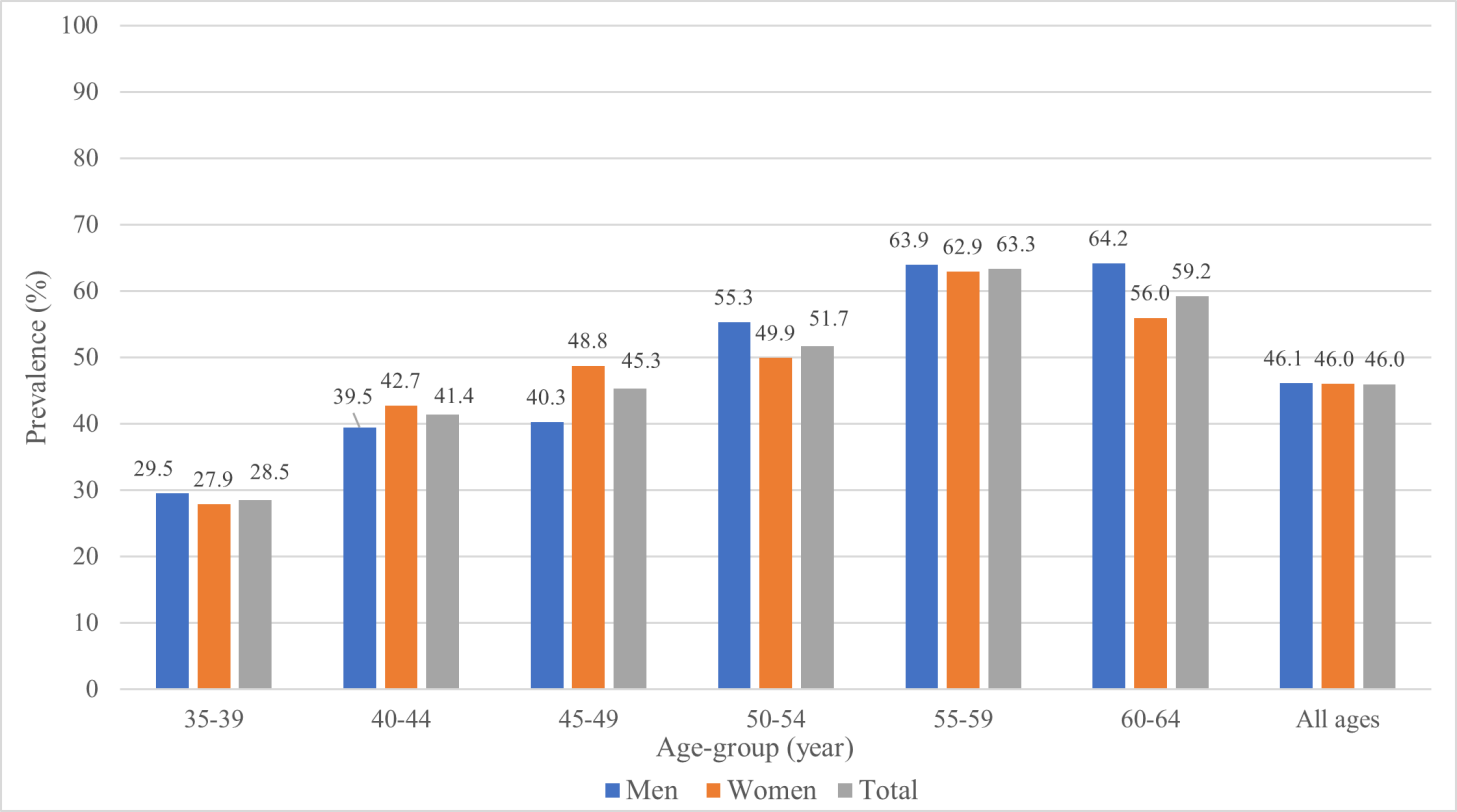
Age-specific prevalence of hypertension among men and women, Namibia DHS 2013.

The prevalence of hypertension increased consistently with age for both sexes until 55–59 years (Fig 1). Adjusted to the WHO world population, the prevalence of hypertension for all ages was 46.0% (95% CI 43.9–48.1). The age-standardized prevalence of hypertension was 46.1% (95%CI 42.9–49.3) for men and 46.0% (95%CI 43.3–48.7) for women.

**Fig 2:**
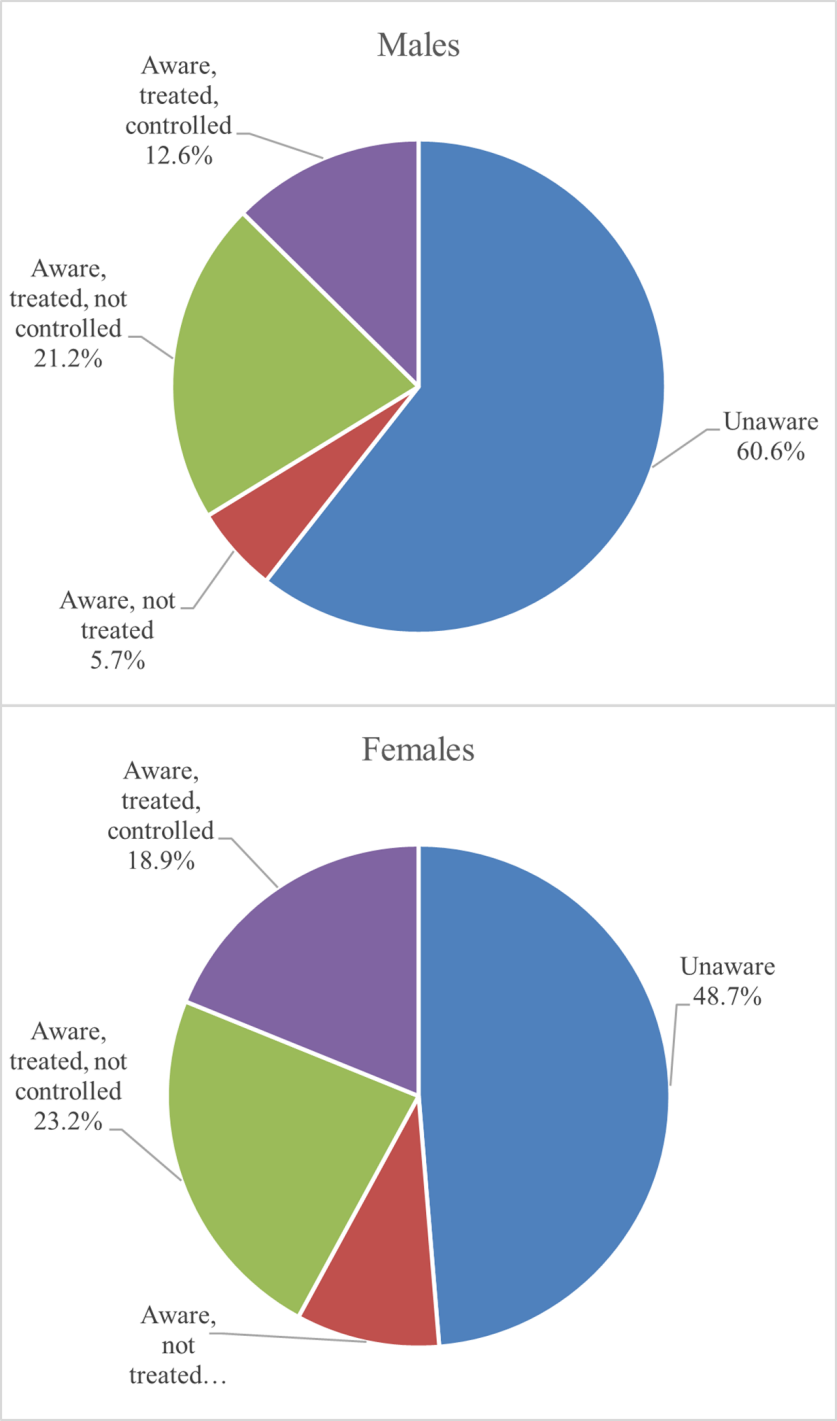
Levels of awareness, treatment and control among men and women with hypertension, Namibia DHS 2013.

Overall, less than half of the study population with hypertension (46.6%) were aware of their condition (Fig 2). While women were more aware of their hypertension than men (51.3% vs. 39.4%; *p*<0.001), there were no significant differences in levels of treatment or control between men and women. About one-third of men (33.8%) with hypertension were on treatment for their condition (compared to 42.1% of women). Among those participants with hypertension, only 12.6% of men and 18.9% of women achieved control. Among both men and women, however, older age-groups were more aware of their hypertension (*p*<0.001) and more likely to be on treatment (*p*<0.05). No significant differences were observed for control rates across age-groups.

### Predictors of hypertension

In fully adjusted analyses (Table 2), age was significantly, positively associated with hypertension in both men and women (*p*<0.001). Similarly, several socio-demographic and metabolic factors were significantly associated with hypertension. Both men and women had significantly increased odds of hypertension if they lived in urban areas or were classified as overweight or obese. For women, the odds of hypertension were higher for those of Herero ethnicity and doubled for those who were diabetic (i.e. had a fasting blood glucose level greater than 7.0 mmol/L) while higher education levels nearly halved the likelihood of hypertension. Being underweight was associated with an approximately 50% lower likelihood of hypertension in both men and women. Interactions between sex and selected predictor variables were also tested but none were found to be statistically significant and are, therefore, not presented.

**Table 2:** Predictors of hypertension prevalence among men and women aged 35–64 years, Namibia DHS 2013.

	Men					Women				
	n = 1,273	No. (%) with hypertension	OR	95% CI	*p*-value	n = 1,795	No. (%) with hypertension	OR	95% CI	*p*-value
**Age (years)**	1,273	556 (44.3)	1.06	1.0–1.1	**<0.001**	1795	813 (44.9)	1.05	1.0–1.1	**<0.001**
**Education**										
No education/preschool only	231	105 (17.3)	[ref]			241	133 (12.9)	[ref]		
Primary	402	171 (30.9)	0.85	0.6–1.2	0.377	638	287 (34.6)	0.70	0.5–0.1	**0.033**
Secondary or higher	640	280 (51.9)	0.80	0.6–1.2	0.241	916	393 (49.1)	0.61	0.4–0.9	**0.009**
**Ethnicity**										
Oshiwambo	463	207 (45.8)	[ref]			758	304 (47.9)	[ref]		
Damara/Nama	230	97 (12.8)	0.76	0.5–1.1	0.128	295	144 (12.7)	0.78	0.6–1.1	0.130
Afrikaans	201	96 (13.4)	0.67	0.4–1.0	0.067	239	128 (10.9)	0.96	0.7–1.4	0.843
Herero	148	69 (11.6)	0.81	0.5–1.2	0.334	179	103 (10.8)	1.47	1.0–2.1	**0.039**
Other	231	87 (16.4)	0.69	0.5–1.0	**0.043**	324	134 (17.7)	0.94	0.7–1.3	0.690
**Residence**										
Rural	659	260 (44.6)	[ref]			981	395 (48.8)	[ref]		
Urban	614	296 (55.4)	1.57	1.2–2.1	**0.002**	814	418 (51.2)	1.63	1.3–2.1	**<0.001**
**Occupational status**										
Not currently working	469	202 (38.2)	[ref]			1137	495 (58.9)	[ref]		
Managerial/professional/technical/clerical	131	67 (12.4)	1.01	0.6–1.6	0.955	215	112 (15.5)	1.26	0.9–1.8	0.199
Sales/services/ industry	362	149 (28.3)	0.73	0.5–1.0	0.063	121	55 (7.0)	1.04	0.7–1.6	0.847
Other	311	138 (21.1)	1.05	0.8–1.4	0.783	322	151 (18.6)	1.16	0.9–1.5	0.312
**Wealth quintile**										
Lowest	196	63 (12.5)	[ref]			332	113 (15.8)	[ref]		
Second	222	96 (16.8)	1.45	0.9–2.3	0.097	343	145 (18.3)	1.23	0.9–1.8	0.235
Middle	261	111 (19.8)	1.18	0.8–1.9	0.465	340	155 (18.8)	1.19	0.8–1.7	0.353
Fourth	309	144 (25.7)	1.07	0.7–1.7	0.787	411	216 (25.4)	1.21	0.8–1.8	0.363
Highest	285	142 (25.3)	0.88	0.5–1.6	0.657	369	184 (21.8)	0.90	0.6–1.5	0.670
**Smoking status**										
Does not currently smoke	882	405 (76.3)	[ref]			1564	687 (87.7)	[ref]		
Smokes cigarettes	309	118 (18.4)	0.88	0.7–1.2	0.412	131	70 (6.0)	1.38	0.9–2.1	0.128
Smokes pipes, cigars, etc.	82	33 (5.3)	0.92	0.6–1.5	0.737	100	56 (6.3)	1.50	1.0–2.4	0.079
**BMI status, kg/m**^**2**^										
Normal weight range	723	280(50.2)	[ref]			699	256 (35.9)	[ref]		
Underweight	186	53(9.3)	0.57	0.4–0.8	**0.004**	156	43 (4.8)	0.63	0.4–1.0	**0.028**
Overweight	228	130(24.5)	2.16	1.5–3.0	**<0.001**	447	207 (24.5)	1.45	1.1–1.9	**0.006**
Obese	136	93(16.0)	3.79	2.4–6.0	**<0.001**	493	307 (34.9)	2.64	2.0–3.5	**<0.001**
**Diabetes**	89	52 (8.4)	1.36	0.8–2.2	0.220	120	85 (8.9)	2.23	1.4–3.4	**<0.001**

DHS = Demographic and Health Survey; OR = odds ratio; CI = confidence interval

Analyses performed separately for males and females

## Discussion

This study examined the prevalence and predictors of hypertension among Namibian adults. The prevalence of hypertension among Namibian adults 35–64 years of age, adjusted to the WHO world population, was approximately 46%, with a similar burden in men and women. In fully-adjusted analyses, men and women had significantly increased odds of hypertension at older age groups, in urban areas, and if they were classified as overweight or obese. For women, the odds of hypertension were also increased for those of Herero ethnicity and those classified as diabetic, while higher education levels were associated with lower odds of hypertension.

The results of this study were consistent with those in the literature reporting high prevalence of hypertension in certain regions of Namibia and relatively lower prevalence of diabetes [15,19,25–28]. Notably, our study revealed a much higher overall prevalence of hypertension in Namibia than in other African countries. These differences can be partly attributed to the older age of the Namibia sample, given that hypertension assumes greater importance with increasing age [9,10]. While our study analyzed data for 35–64 year old adults, other studies have estimated hypertension prevalence among those aged 15 and older [8,12] or 20 years and older [4,13]. Nonetheless, the estimates reported here are similar to hypertension prevalence estimates in Punjab, India (40%) [29] and in the USA among African Americans (44%) [30].

Findings from this study are also in line with the evidence of urban environments, advancing age and increased BMI–namely, overweight and obesity–as important drivers of hypertension in SSA populations [1,2,4,8–10,12]. The increased odds of hypertension among women of Herero ethnicity are also consistent with an earlier study documenting essential hypertension as common in Herero populations [31]. Results of this study also support evidence of low levels of awareness of hypertension among Namibian adults, in addition to significant differences across sex and age-groups [13,15,32]. This finding may be indicative of education as an important area for intervention in national efforts to address disease prevention and control, given lower rates of hypertension among more educated women, in addition to overall low knowledge and self-perceived risk of NCDs, which were found by a previous study of formal sector employees across 13 industries in Namibia [27].

The female preponderance in overweight and obesity in addition to the increased odds of hypertension among overweight and obese men and women suggest the need for culturally-appropriate, nutritional interventions targeting optimal weight management. This need is especially pressing given the established relationship between overweight/obesity and cardiovascular disease and diabetes, as well as the increasing trend in overweight/obesity in all African countries, particularly among women in Southern Africa [6,33,34]. Other vulnerable groups, including persons living urban areas, older age-groups and uneducated women, need to be considered in the development of behavior change strategies and other public health interventions to better mitigate the risk of hypertension and the potentially damaging burden of NCDs. Relatively high levels of pre-hypertension combined with low levels of awareness among those with hypertension suggest increased vulnerability of this population and the need for increased sensitization regarding the dangers associated with raised blood pressure levels.

From a program perspective, the results of this study call for effective management and prevention strategies to curb disease progression and complications. In response to the increasing morbidity and mortality burden from NCDs, the MoHSS in Namibia has been strengthening its national systems response, developing policies, passing legislation and launching several initiatives to raise awareness, promote healthy behaviors and support increased prevention throughout neighborhoods, schools and communities [25,35,36]. Specifically, the National Health Policy Framework 2010–2020 outlines several strategic directions adopted by the Namibia government in response to the rising NCD epidemic, including behavior change communication and health promotion through community dialogue, health system strengthening, increased surveillance of NCD risk factors, and normalization of NCD screening [37]. In targeting prevention and management strategies to populations at greater risk, comorbid hypertension and diabetes may prove an important area for investigation and intervention given similar underlying pathophysiological disease mechanisms and moderate burden of diabetes among men and women in this population.

Finally, since in many African contexts, NCDs have not simply displaced communicable diseases as the primary cause of morbidity and mortality, future research should also explore the double burden of communicable diseases & NCDs, as this occurrence has significant implications for health systems and health outcomes, and can signify the need for new policies, investments in health infrastructure and integrative services [5]. Future work should also explore concurrent use of both traditional remedies and prescription medication among men and women as this may have significant implications for intervention design, patient education strategies and adverse drug interactions and outcomes.

### Limitations

There are several study limitations to be considered. First, coverage rates for blood pressure measurements among Namibian men and women were low at 71.2% and 80.7%, respectively. These coverage rates were lower than other DHS African countries such as the 2009 Lesotho DHS (96% for both men and women) and the 2014 Ghana DHS (women: 99.6%; men: 99.7%) [22,38]. Interestingly, coverage rates in Namibia were similar to those for the 2007 Ukraine DHS where 74% of men and women consented to have their blood pressure measured. Aside from those who were missing information, most commonly cited reasons for non-coverage of blood pressure measurements in the Namibia sample were absence from the home (30.1%) and refusals (17.4%). Some populations are reluctant to have their blood drawn [39,40] and it is unclear whether respondent fatigue or the sequencing of blood draws may have affected response rates (i.e. blood sampling in children for anemia testing was performed prior to blood pressure measurements). The high levels of nonresponse among Namibian women and, in particular, Namibian men could potentially bias the hypertension prevalence estimated reported here.

Secondly, while use of the 2013 NDHS–as the first national survey in Namibia to include biomarker measurements of blood pressure and fasting blood glucose [18]—is a major strength of this study, the sample of respondents from whom objective assessments were measured was limited to those 35–64 years of age, narrowing the representativeness of estimates and precluding exploration of the burden and distribution of disease across younger and older age-groups. Furthermore, other important predictors of hypertension risk, such as alcohol consumption, physical inactivity, and fruit and vegetable consumption, were not investigated here since these data were only collected in a sub-sample of respondents 15–49 years of age. These lifestyle-linked factors are often widely distributed in the population and may be important in understanding vulnerability and predicting risk [5,14,41–44]. This is particularly true for the Namibia population given evidence that Herero women, for example, tend to have elevated BMI levels, be less physically active, and consume high-carbohydrate diets with low intakes of vegetables [31]. Future studies should consider investigation of the role of the behavioral factors in understanding morbidity and mortality from hypertension and other NCDs.

## Conclusion

The prevalence of hypertension among Namibian adults is high and associated with metabolic- and socio-demographic factors. Future research examining trends and comorbidity with other infectious and/or chronic diseases is needed, to better understand the disease burden in this population and target resources to optimize disease prevention, management and control.
